# Head and neck reconstruction using infrahyoid myocutaneous flaps

**DOI:** 10.1590/S1516-31802006000500007

**Published:** 2006-09-07

**Authors:** Alfio José Tincani, André Del Negro, Priscila Pereira Costa Araújo, Hugo Kenzo Akashi, Flávia da Silva Pinto Neves, Antônio Santos Martins

**Keywords:** Head and neck neoplasms, Surgical flaps, Reconstructive surgical procedures, Neck muscles, Rehabilitation, Câncer de cabeça e pescoço, Retalhos cirúrgicos, Procedimentos cirúrgicos reconstrutivos, Músculos do pescoço, Reabilitação

## Abstract

**CONTEXT AND OBJECTIVE:**

The use of pedicled myocutaneous flaps in head and neck reconstruction is widely accepted. Here we describe our experience with infrahyoid flaps (IHFs) employed to cover surgical defects in the oral cavity and oropharynx in patients with benign and malignant tumors. The aim was to evaluate the success rate for infrahyoid myocutaneous flap procedures performed at a single institution.

**DESIGN AND SETTING:**

Retrospective study, at the Head and Neck Surgery Service, Unicamp.

**METHODS:**

Fourteen IHFs were used to reconstruct surgical defects in eleven men (78.5%) and three women (21.5%) with a mean age of 66.4 years. The anterior floor of the mouth was reconstructed in nine patients (64.2%), the base of tongue in three (21.4%), the lateral floor in one (7.1%), and the retromolar area (7.1%) in one. Thirteen patients (92.8%) had squamous cell carcinoma (SCC) and one (7.2%) ameloblastoma. The disease stage was T3 in eight (61.5%) of the SCC cases and T4 in five (38.5%).

**RESULTS:**

No patient presented total flap loss or fistula. The most common complication was epidermolysis, which delayed the beginning of oral ingestion. The patients with SCC received postoperative radiotherapy without major consequences to the flap.

**CONCLUSION:**

IHF is a safe and reliable procedure for reconstructing head and neck surgical defects. Due to its thinness and malleability, its use for oral cavity and oropharynx defects provides favorable cosmetic and functional outcomes. Complications, when present, are easy to manage.

## RESUMO

**CONTEXTO E OBJETIVO:**

O uso de retalhos miocutâneos pediculados para reconstrução cirúrgica na região de cabeça e pescoço (RCP) é consagrado. Apresentaremos a experiência com o uso do retalho infrahiódeo (RIH) em reconstrução de defeitos cirúrgicos na cavidade oral e orofaringe em portadores de tumores benignos e malignos. O objetivo foi avaliar o índice de sucesso do RIH em defeitos da cavidade oral em uma única instituição.

**TIPO DE ESTUDO E LOCAL:**

Estudo retrospectivo, no Serviço de Cirurgia de Cabeça e Pescoço, Universidade Estadual de Campinas (Unicamp).

**MÉTODOS:**

Foram utilizados 14 RIH para reconstrução em RCP em 11 homens (78,5%) e 3 mulheres (21,5%). Em nove (64,2%) pacientes, a reconstrução foi de assoalho oral anterior, três (21,4%) de base de língua, um (7,1%) de assoalho lateral e um de trígono retromolar (7,1%). O carcinoma espinocelular (CEC) foi neoplasia presente em 13 deles (92,8%) e um (7,2%) ameloblastoma de mandíbula. O estádio era T3 em oito (61,5%) e T4 em cinco (38,5%) dos casos de CEC.

**RESULTADOS:**

Não houve caso de perda total do retalho ou fístula. A complicação mais comum foi epidermólise, retardando o início da ingestão oral. Pacientes com CEC receberam radioterapia pós-operatória sem conseqüências para o retalho.

**CONCLUSÃO:**

O RIH é um retalho seguro e confiável para o cirurgião de cabeça e pescoço na reconstrução na RCP. Devido à espessura e maleabilidade, sua utilização para reconstrução de defeitos da cavidade oral e orofaringe proporciona bom aspecto cosmético e funcional. Complicações são de fácil manuseio.

## INTRODUCTION

The use of myocutaneous flaps is now a well-established alternative for reconstructing surgical defects in the head and neck.^1-6^ Despite the evolution of free flaps with microsurgical anastomoses, many axial pedicled flaps are widely used because of their ease of harvesting, the reliability of the pedicle, the experience of most surgeons in using them and the non-availability of surgical teams able to perform free flap procedures.

First described by Wang et al. in 1986^1^ and called the infrahyoid flap (IHF), these flaps were proposed for the reconstruction of defects following resections of the oral tongue and, subsequently, for more complex defects of the oral cavity, oropharynx and facial skin. The flap is comprised of skin from the cervical region and the strap muscles, and its pedicle is based on the superior thyroid vessels.

## OBJECTIVE

The objective of this article was to describe our experience with 14 patients with head and neck neoplasms, in whom infrahyoid myocutaneous flaps were used for reconstruction.

## MATERIALS AND METHODS

Between January 1994 and December 2004, 14 patients with neoplasms of the head and neck underwent surgical resection of their lesions at the Head and Neck Surgery Service of the Department of Surgery, Universidade Estadual de Campinas (Unicamp), Brazil. Patients with prior cervical surgical procedures were excluded from the study, as were patients with an ipsilateral scar on the flap area, advanced atherosclerosis that might have impaired the blood flow in the flap, or neck metastasis that required radical neck dissection (N2 or N3). Thirteen (92,8%) out of the 14 patients included over the period had squamous cell carcinoma (SCC) and one

(7.2%) had ameloblastoma. The disease stage was T3 in eight (61.5%) of the SCC cases and T4 in five (38.5%). All of these patients had their surgical defects reconstructed using IHFs. Eleven of them were men and three were women, with ages ranging from 47 to 80 years (mean age of 66.4 years).

Excessively hairy skin was a relative contraindication for flap use. Previous irradiation of the neck was also a contraindication for the use of IHFs, as stated by other authors.^3,6^

**Figure 1:**
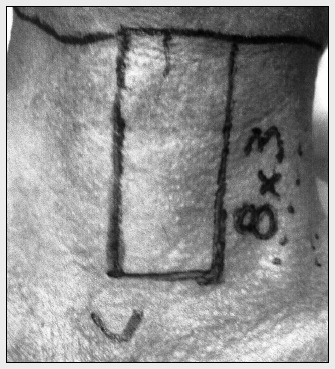
Pedicle flap composed of infrahyoid strap muscles that was used for surgical reconstruction of the head and neck.

The infrahyoid strap muscles were used for this flap, including the sternohyoid, sternothyroid and the inferior belly of the omohyoid. The superior portion was vascularized by the superior thyroid artery and its branches, and the inferior third by the inferior thyroid artery. According to Eliachar et al.,^2^ this blood supply is segmental but presents significant anastomoses between the main superior and inferior branches, thereby permeating the entire musculature. Venous drainage takes place through the inferior thyroid vein, which drains directly into the internal jugular vein or through the anterior ipsilateral jugular vein (Figure 1).

According to Wang et al.,^1^ sensorimotor innervation comes directly from branches of the cervical loops of C1 and C2 (upper half) and C2 and C3 (lower half), through nerve anastomoses.

**Figure 2:**
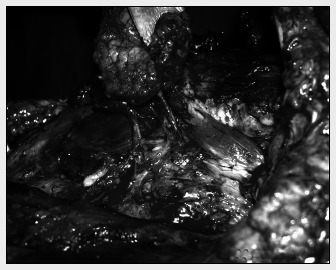
The miocutaneous flap was harvested by cutting the skin in a cranialcaudal direction, in a paramedian or central location.

The flap was harvested by cutting the skin in a cranial-caudal direction, in a paramedian or central location, depending on the site to be reconstructed (Figure 2). Care was taken not to go beyond the contralateral boundaries, thus avoiding damage to local micronutrition.

After the rectangular island of skin was constructed, in continuity with the subcutaneous tissue and the abovementioned muscles, the unit was separated by fine dissection of the surgical capsule of the thyroid gland, in the plane of the superficial layer of the deep cervical fascia. Its size ranged from 10 to 14 centimeters,^1,3^ depending on the defect to be corrected. The thyrohyoid muscle was preserved and remained *in situ* in order to protect the superior laryngeal nerves and laryngeal vessels.^4^ As the flap was harvested, even greater care was taken in dissecting the upper third. The vascular pedicle could be identified easily and was clearly visible when dissection occurred in the medial-lateral direction. After identifying the pedicle, it was skeletonized by ligating its collateral branches (superior laryngeal artery, infrahyoid artery and cricothyroid artery, and their branches to the sternocleidomastoid muscle; and the collateral veins) and expanding its rotation arch,^5^ which could be up to 15 centimeters in length.^1^ Special attention was given to the dissection of the superior laryngeal nerve, which must always be preserved. As stated by Wang et al.,^1^ the collateral vessels should be maintained whenever possible.

The island of skin on the flap was sutured to the subjacent musculature to avoid sliding between its layers, which could injure the *perforans* vessels and thus compromise the microvasculature. The anterior jugular vein was ligated in its inferior and superior portions and the muscles were deinserted in this same direction, from the sternum toward the hyoid bone, where they were sectioned. The third branch of the cervical loop had to be sacrificed, thereby providing the flap with a broader rotation arch.

After adequate hemostasis, the skin of the donor area was closed primarily without difficulties.

The flap was used to reconstruct defects in the floor of the mouth and the oropharynx. Nine cases were in the anterior floor of the mouth (all adjacent to or directly involving the jaw), one affected the lateral floor, three were at the base of tongue and one was in the retromolar area. All these lesions, except for one ameloblastoma, had first been clinically staged as T3 or T4 (Table 1).

**Table 1:** Description of the series of cases of head and neck reconstruction using infrahyoid myocutaneous flap

Patient	Tumor site	Flap size (cm)	Function	Complications	Oral diet (days)	Feeding tube (days)	Recurrence
1	FOM T3	8 × 4	Good	No	6	7	No
2	FOM T4	8 × 5	Fair	No	7	8	Yes
3	Tongue base T3	9 × 4	Fair	Donor site dehiscence	10	12	Yes
4	RMT T4	8 × 4	Good	No	5	6	No
5	FOM T4	8 × 3	Good	Skin paddle necrosis	9	10	No
6	Tongue base T4	8 × 4	Fair	Skin paddle necrosis	10	11	Yes
7	FOM T3	8 × 3	Good	No	6	5	No
8	FOM T3	8 × 4	Good	No	7	8	No
9	FOM T4	8 × 3	Good	No	8	8	No
10	Tongue base T4	8 × 4	Fair	Skin paddle necrosis	9	10	Yes
11	FOM T4	9 × 4	Good	Post RT retraction	6	7	No
12	FOM T4	10 × 4	Good	Skin paddle necrosis	9	10	No
13	FOM T4	10 × 4	Good	No	7	8	No
14	Ameloblastoma	8 × 5	Good	No	5	6	No

FOM = floor of the mouth; RMT = retromolar trigone; RT = radiotherapy.

The variables analyzed were: size of the flap, stage of the tumor upon diagnosis, primary site and functional results.

All patients (except the one case of ameloblastoma) received postoperative radiotherapy, with individualized doses for each case, ranging from 5000 to 7000 cGy. Supraomohyoid neck dissection was performed on all cases with malignant tumors (all N0 upon diagnosis), and the dissection was bilateral when the lesion was adjacent to or crossed the midline.

The flap was used with primary closing of the donor area in all cases. The skin paddle was rectangular and paramedian in all cases, as postulated by Wang et al.,^1^ with size varying according to the area to be re constructed (Table 1).

Motor innervation was preserved in all cases, and tracheostomy was performed in 13 patients.

Clinically palpable cervical lymph nodes located at levels I and II and no larger than two centimeters (N1) were considered for selective neck dissection, with no prejudice to the venous draining of the flap.

## RESULTS

No case resulted in total loss of the flap and only four cases (28.5%) showed partial loss through epidermolysis, which did not affect the final result. Damage to the cutaneous portion occurred within the first three days following reconstruction and there was no loss of the muscular portion of the flap. There was one case of retraction of the flap caused by radiotherapy. However, no alteration of the functional results of the surgery was observed.

No cases of salivary fistulas were seen, nor were there any early or late hemorrhagic or infectious complications.

Regarding the donor area, only one patient showed partial dehiscence of the suture line, which was resolved with local care and produced very satisfactory final aesthetic results.

The nasoenteral feeding tube was removed on average eight days after surgery and oral nutrition was introduced on approximately the seventh day. The patients showed full capacity for oral ingestion of food on the twelfth day following surgery.

The patients with SCC received postoperative radiotherapy without major consequences to the flap. To date, four patients have shown local-regional recurrence, even after adjuvant treatment.

With regard to swallowing and speaking capacity, four patients required postoperative speech therapy. This evaluation was subjective and based on the criteria of "good", "intermediate" or "poor" capacity to produce phonemes, in comparison with the patient's corresponding presurgery ability. The evaluation was considered reliable since the patients did not present any complicating factors that might influence their speaking ability, such as radiotherapy or prior head or neck surgery. Nevertheless, both the anatomical location of the surgical defect and the harvesting of the flap negatively affected functional results.

## DISCUSSION

A variety of flaps are available for reconstructing surgical defects in the head and neck, including the pectoralis major, deltopectoral, trapezius and platysma, among others, and each has its own advantages and disadvantages. The ideal flap should be thin, pliable, hairless, easy to harvest and reliable and should have the capacity for implementation in a singlestage procedure.

Although the most commonly used flap is the pectoralis major, the IHF is thinner, lies close to the surgical defect and, based on our current experience, is very reliable. Other advantages are that it is easy and quick to harvest; there is no need to reposition the patient; there is primary closure of the donor area; and there are few or no cosmetic sequelae. The primary indications for using IHFs are intraoral, pharyngeal and parotid region skin defects.^1-3,5,6^

We believe that it is essential to suture the muscles to the skin, in order to attach them and thus prevent slipping. Likewise, there needs to be careful handling both in harvesting and elevation. These steps are likely to avoid the epidermolysis that occurred in several of our cases. Our success rate approached 100% and was comparable to larger series with similar complications.^1,3,5,6^

As recommended by the majority of authors, only patients who had not undergone previous cervical radiotherapy were selected for IHFs.^3,6^

In the cases with loss of the cutaneous portion of the flap, this probably occurred for the same reasons as described by Wang,^1^ that is, because of a deficit in venous drainage and consequent flap congestion.

Preserving the motor innervation in all cases provides greater symmetry, volume and mobility to the flap, thereby reducing atrophy and improving the functional results. This allowed for subsequent positive functional results regarding swallowing and speaking in our series.

Tumor recurrence was due to the advanced stage of the disease at diagnosis, and this finding was similar to what has been described by other authors.^4^

## CONCLUSION

The infrahyoid myocutaneous flap procedure is simple for a head and neck surgeon familiar with the anatomy of this region to perform. In our experience, there were positive aesthetic and functional results, and the complications were easy to manage. The patients were, however, carefully chosen so as to respect the contraindications.

Among the most important advantages of this type of flap are: the more appropriate thickness and coloring of the skin than in the material obtained from the usual flaps (the type used here was thinner and more pliable than the skin of the thoracic wall or the forearm); the head and neck surgeon's familiarity with the anatomical region; the proximity of the donor area to the reconstruction site; and ever-present possibility of primary closure. In addition, these flaps present satisfactory cosmetic appearance and are well accepted by patients.
